# Perseverative thinking, threat interpretation bias, and emotional reactivity as mediators between adverse childhood experience domains and psychopathology: A longitudinal mediation study in a cohort of Swiss emerging adults

**DOI:** 10.1016/j.ijchp.2025.100654

**Published:** 2025-12-11

**Authors:** Jeannette Brodbeck, Sofia Jacinto, Lina Stallmann, Neela Vetsch, Simon Marmet, Sharon A. Neufeld

**Affiliations:** a School of Social Work, University of Applied Sciences and Arts Northwestern Switzerland, Olten, Switzerland; b Department for Clinical Psychology and Psychotherapy, University of Bern, Bern, Switzerland; c Centre for Psychological Research and Social Intervention (Cis-IUL), Instituto Universitário de Lisboa (Iscte-IUL), Lisboa, Portugal; d Department of Psychiatry, University of Cambridge, Cambridge, United Kingdom

**Keywords:** Adverse childhood experiences, Psychopathology, Emotion regulation, Social information processing, Longitudinal mediation, Peer victimisation, Emerging adulthood

## Abstract

Adverse Childhood Experiences (ACEs) are robust predictors of negative mental health outcomes and psychosocial difficulties, yet the psychological mechanisms linking ACEs to later psychopathology remain only partially understood. Drawing on a three-wave longitudinal study of Swiss emerging adults (*N* = 1934), we conducted longitudinal mediation analyses to examine emotional processing (emotional reactivity, perseverative thinking) and social information processing (threat interpretation bias, rejection sensitivity) as pathways from ACEs to psychopathology. Factor analyses identified three distinct ACE domains: family maltreatment, peer victimization, and sexual abuse. By modelling these domains simultaneously, we accounted for their frequent co-occurrence and isolated their unique contributions. Family maltreatment and peer victimization independently were associated with heightened psychopathology and difficulties with emotional processing and social information processing at Wave 1. Furthermore, both adversity domains also predicted persistent elevations in these domains over time, even after controlling for baseline levels and sociodemographic variables. Longitudinal mediation analyses revealed that family maltreatment and peer victimization both predicted psychopathology via perseverative thinking, threat interpretation bias, and emotional reactivity. Sexual abuse, in contrast, showed weaker or delayed associations with psychopathology and operated primarily through threat interpretation bias. Rejection sensitivity, while associated at the bivariate level, did not mediate longitudinal effects. Findings support and extend McLaughlin’s Model of Mechanisms Linking Childhood Trauma to Psychopathology by identifying distinct mediational pathways from specific ACEs to psychopathology. These distinct pathways underscore the relevance of personalized and mechanism-based treatment planning based on the ACEs experienced.

Adverse childhood experiences (ACEs) are associated with an increased risk for a range of negative outcomes, including mental health conditions and social and behavioural difficulties, typically with small to moderate effect sizes (Hashim et al., 2024, 2025; Kim & Royle, 2025; Kuzminskaite et al., 2021; Lee et al., 2025). Beyond the traditional categories of ACEs such as child maltreatment and household dysfunction, emerging evidence indicates that peer victimization and bullying function similarly to other ACEs, with several studies demonstrating significant associations with adverse adult social, physical and mental health outcomes (Halliday et al., 2021; Karatekin & Hill, 2019; Li et al., 2024; Rijlaarsdam et al., 2021; Trompeter et al., 2024).

While the associations between ACEs and adverse outcomes are well documented, less is known about the psychological mechanisms that link ACEs with psychopathology later in life. Understanding these mechanisms will expand our knowledge of the interaction between the social environment and developmental processes in the aetiology of psychopathology. Moreover, identifying malleable mechanisms as targets for interventions—and gaining a nuanced understanding of how they contribute to psychopathology—can inform the development and optimisation of focused clinical care and preventive strategies (McLaughlin et al., 2020). However, ACEs encompass a wide range of events—such as emotional abuse, physical neglect, sexual violence, household dysfunction including witnessing violence, and peer victimization—that often co-occur. Thus, there is a need for research to elucidate whether distinct adversities share common pathways to psychopathology or rely on unique mechanisms.

Drawing on several decades of research, McLaughlin proposed the Transdiagnostic Model of Mechanisms Linking Childhood Trauma to Psychopathology (McLaughlin et al., 2020). This model outlines key mechanisms through which threat-related ACEs increase vulnerability to internalising and externalising psychopathology. The model identifies three core pathways affected by ACEs: (1) emotion processing, including heightened emotional reactivity and impaired emotion regulation; (2) social information processing, characterized by enhanced threat detection and hostile attribution bias; and (3) accelerated biological aging. Disruptions in these mechanisms are implicated in the development of both internalizing and externalizing transdiagnostic psychopathologies whereas social support is posited as a protective transdiagnostic factor.

## Emotion processing mechanisms linking ACEs to psychopathology

Emotion regulation deficits — including high emotional reactivity and perseverative thinking —have been consistently identified as risk factors for psychological disorders as well as mediators of the impact of ACEs on poorer psychosocial and mental health outcomes.

***Emotional reactivity*** refers to the intensity and duration of emotional responses as well as the ease with which emotions are triggered by internal or external stimuli. It encompasses both the immediate activation of an emotion and the time required to return to baseline. Individuals with a history of ACEs often exhibit heightened emotional reactivity, reflected in amplified stress responses and difficulty regulating affective states in childhood, adolescence and adulthood (Lavi et al., 2019; Potter et al., 2025). A recent cross-sectional study among emerging adults found differential associations between types of ACE and emotional reactivity (Ryder et al., 2025). Emotional neglect and emotional abuse were positively associated with emotional reactivity, whereas physical neglect, physical abuse, and sexual abuse were not. Beyond being affected by ACEs, elevated emotional reactivity has been shown in cross-sectional, experimental, and longitudinal studies to mediate the relationship between ACEs and both internalising and externalising symptoms in adolescence (McLaughlin et al., 2020). Nevertheless, longitudinal studies in large epidemiological samples remain rare.

***Perseverative Thinking*** is characterized by repetitive, intrusive, and difficult-to-control thoughts focused on negative experiences, persistent worry, or perceived threats. This construct includes cognitive patterns such as rumination—excessive focus on past distressing events—and chronic worry about uncertain future outcomes (Ehring & Watkins, 2008; Hallion et al., 2022). Perseverative thinking is recognized as a transdiagnostic cognitive process associated with depression, anxiety, and broader psychological dysfunction (Ehring & Watkins, 2008; Moulds & McEvoy, 2025). Meta-analyses and systematic reviews have substantiated both the association between ACEs and perseverative negative thinking and the mediating role of such thinking in the link between ACEs and diverse psychopathological and psychosocial outcomes in adulthood (Mansueto et al., 2021; Miu et al., 2022). When examining the role of specific types of ACEs in relation to rumination, consistent associations have been found for emotional and physical abuse, whereas findings for emotional and physical neglect, as well as sexual abuse, remain mixed (Gao et al., 2017).

### Social information processing mechanisms linking ACEs with psychopathology

Social information processing constructs *—* including social rejection sensitivity and interpretation biases — have been identified as transdiagnostic risk factors for psychological disorders (Gao et al., 2017; Lavigne et al., 2024).

***Rejection sensitivity*** describes the tendency to anxiously expect, readily (and unjustifiably) perceive, and react intensively to social rejection (Downey & Feldman, 1996). A recent meta-analytic review confirmed that ACEs were associated with higher rejection sensitivity in adulthood. Although all forms of child maltreatment predicted higher rejection sensitivity, the effect was stronger for emotional abuse than for physical abuse (Gao et al., 2024). In contrast, a cross-sectional study with 311 emerging adults (Euteneuer et al., 2024) found that whereas emotional abuse and neglect had unique associations with rejection sensitivity, physical abuse, physical neglect, and sexual abuse did not. Despite evidence that rejection sensitivity follows ACEs (Euteneuer et al., 2024; Gao et al., 2024) and predicts later psychopathology (Gao et al., 2017) studies that examined rejection sensitivity as a mediator of the association between ACEs and psychopathology are rare.

***Threat interpretation bias***—a form of negative interpretation bias—refers to the biased perception, hypervigilance, and over-generalisation of cues that may signal danger (Boffa et al., 2018). Experimental studies suggest that threat related information processing is a transdiagnostic mechanism linking ACEs with psychopathology (Weissman et al., 2019). Minihan et al. found that both increased rejection sensitivity and negative interpretation bias independently and partially mediated the association between heightened perceptions of parental rejection and later emotional disorder symptoms (Minihan et al., 2023).

In summary, recent research has identified emotion processing as a key candidate mechanism and confirmed that several facets of emotion processing mediate the association between ACEs and later psychosocial functioning. However, greater clarity is needed regarding how the type of ACE may moderate associations with emotion processing. A separate body of literature—often using experimental designs—highlights social-information-processing factors, including attention and interpretation biases and rejection sensitivity, as additional candidate mechanisms. However, operational definitions of threat interpretation bias vary considerably and to the best of our knowledge, no large-scale epidemiological study has examined rejection sensitivity and threat interpretation bias as mediators for the association between ACEs and psychopathology. Furthermore, no longitudinal study has simultaneously examined emotion processing and social information processing mechanisms in predicting psychopathology during (emerging) adulthood, nor compared their relative contributions to the long-term impact of ACEs. Moreover, as different ACEs may operate through distinct mechanisms, there is a lack of studies that investigate whether different types or domains of ACEs share pathways or whether specific mechanisms are unique to particular ACEs. This not only deepens our understanding of how ACEs exert their effects but also helps identify potential targets for personalised interventions.

### The present study

The present study aims to contribute to the existing body of knowledge in three distinct ways. First, it uses a comprehensive assessment of ACEs—including physical and emotional abuse and neglect, sexual abuse, witnessing domestic violence, and peer victimization—to examine psychological mechanisms linking ACE domains to psychopathology in a large population-based sample of emerging adults. Emerging adulthood (ages 18–25) is marked by social and environmental transitions: gaining financial independence, progressing in education or employment, forming romantic relationships, and leaving the parental home (Arnett, 2015). Within the context of ACEs, emerging adulthood offers a window of opportunity to redirect maladaptive trajectories toward healthier paths but also poses challenges to psychosocial adaptation including the risk of consolidation of problems. Between ages 18 and 25, the proportion of individuals who have experienced the onset of at least one mental disorder rises from 48.4 % to 62.5 % (Solmi et al., 2022). Insights into these trajectories and their underlying mechanisms can inform interventions to prevent or mitigate the negative effects of ACEs during this critical life stage.

Second, drawing on the Transdiagnostic Model of Mechanisms Linking Childhood Trauma to Psychopathology (McLaughlin et al., 2020), the present study integrates candidate mediators from the domains of emotion regulation and social-information processing, comparing their specific longitudinal contributions to the link between ACEs and psychopathology in emerging adulthood. Specifically, we analyse the mediating roles of (a) heightened emotional reactivity and perseverative thinking—as indicators of poor emotion regulation—and (b) increased threat interpretation bias and social rejection sensitivity—as components of social-information processing. We hypothesise that ACEs will be associated with increased emotional reactivity, rejection sensitivity, perseverative thinking, and threat-interpretation biases; and in turn, elevated levels of these constructs will predict higher subsequent psychopathology. Accordingly, we expect these variables to mediate the longitudinal relationship between ACEs and psychopathology during emerging adulthood. Using longitudinal dual-process simplex path modelling (Goldsmith et al., 2018), we examine whether the mediation effects are: (a) *proximal*, i.e. have a short-term early effect, (b) *distal*, i.e. have a delayed effect and become relevant later, or (c) represent *sustained* effects, i.e. unfold across time through a mediator that continues to exert influence across waves, affecting an outcome at a later wave. We further compare the relative strength of these associations using a multiple mediation model that includes all mediators simultaneously.

**Fig. 1:**
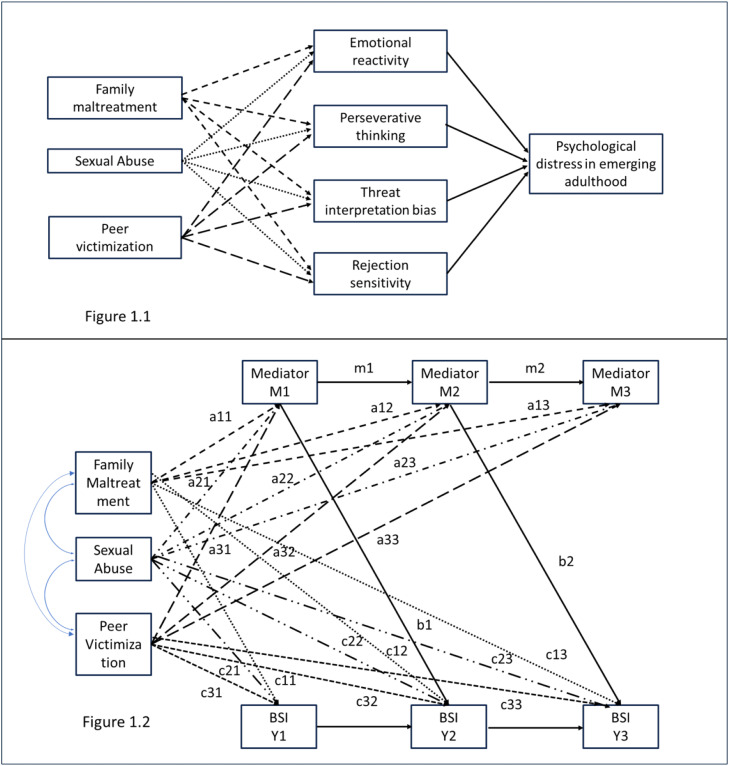
(a) Theoretical schematic of the hypothesised parallel mediation model with ACE domains predicting psychological distress in emerging adulthood via all four mediators (b) Longitudinal dual process simplex model for one single mediator and lagged b paths, depicting all theoretical direct paths. Contemporaneous residual covariance paths not shown for simplicity. BSI: Psychological distress.

Third, based on a factor model of seven ACE types, we test whether distinct ACE domains - (1) family maltreatment, including physical/emotional maltreatment and witnessing domestic violence, (2) peer victimization, and (3) sexual abuse - differ in their associations with psychopathology and the proposed mediators. Given the frequent co-occurrence of adversities, it is crucial to include all ACE domains in the same analyses to assess their unique independent contributions. Fig. 1a illustrates the theoretical mediation model. In sum, this study extends existing knowledge by evaluating candidate mediators from the domains of emotion processing and social information processing, and by quantifying the differential effects of ACE domains on these mechanisms, thereby contributing to a better understanding of how ACEs shape psychopathology in emerging adulthood.

## Methods

### Sample

The Swiss Federal Statistical Office provided a random sample of postal addresses of emerging adults aged 18 to 21 years living in private households in German-speaking regions of Switzerland, see (Brodbeck et al., 2022). A total of 15,000 individuals were contacted by postal mail and invited to participate in an online survey. As the response rate was lower than expected and an adequate sample size was required for the intervention component of the FACE project, an additional 167 participants were recruited via universities, teacher training colleges, and military training schools mandatory for men. In total, 2439 individuals from the random sample and 167 from the additional recruitment pool provided electronic informed consent and initiated the survey. Participants completed the online self-report questionnaires at Wave 1 (W1) in the winter of 2021/22, with annual follow-ups at Wave 2 (W2) and Wave 3 (W3). The final analytic sample for this study consists of 1934 participants who completed at least 90 % of the CTQ and the MACE items (*n* = 1982) and who did not show unlikely answer patterns such as straight-lining or completion time below 10 min (*n* = 48).

This study was reviewed and authorized by the Ethical Committee of Northwestern and Central Switzerland (BASEC number 2021–01,204) and was pre-registered at clinicaltrials.gov (NCT05122988). This work was funded by the Swiss National Science foundation [Grant Number SNF 100010_197731, JB].

## Measures

### Adverse childhood experiences

*Child maltreatment* was assessed using the Childhood Trauma Questionnaire (CTQ; Bernstein et al., 1998; Häuser et al., 2011). The CTQ includes five subscales measuring mostly family-related physical abuse, emotional abuse, sexual abuse, physical neglect, and emotional neglect, referring to experiences occurring before the age of 18. Each subscale consists of five items rated on a 5-point Likert scale (1 = “never”, 2 = “rarely”, 3 = “sometimes”, 4 = “often” and 5 = “very often”). Reverse coded items were recoded so that on each item a score of 1 represents the best outcome and 5 the worst. Internal consistency and structural validity of the CTQ as well as the test-retest reliability were confirmed in meta-analyses and single studies, with the physical neglect subscale providing lower psychometric properties (Badenes-Ribera et al., 2024; Georgieva et al., 2023; see also Dudeck et al., 2015; Karos et al., 2014) for the German version of the CTQ). In this sample, Cronbach alpha was excellent for the emotional neglect (Cronbach’s α = 0.91) and sexual abuse subscales (Cronbach’s α = 0.90), good for the emotional abuse (Cronbach’s α = 0.88) and the physical abuse subscales (Cronbach’s α = 0.80), and questionable for physical neglect subscale (Cronbach’s α = 0.60).

*Peer victimization*—including verbal, physical, and sexual abuse by peers (6 items)—as well as *Witnessing domestic violence* (5 items) were both assessed using items adapted from the German version of the Maltreatment and Abuse Chronology of Exposure (MACE) scale (Isele et al., 2014; Teicher & Parigger, 2015). The MACE showed a good reliability for the subscales and excellent test-retest reliability (ibid). Response options for the MACE items were aligned with the CTQ to ensure consistency across measures. In this sample, Cronbach alpha was good for peer victimization (Cronbach’s α = 0.83) and acceptable for witnessing domestic violence sample (Cronbach’s α = 0.76).

*Psychopathology* as outcome variable was assessed using the global severity index of the Brief Symptom Inventory −18 (BSI-18; Spitzer, 2011), a well-established self-report instrument designed to evaluate general psychological distress in population-based samples (Franke et al., 2017). The BSI-18 includes three dimensions—depression, anxiety, and somatization—each represented by six items rated on a 5-point Likert scale ranging from 1 = “*not at all* “to 5 = “*extremely*” and the total BSI-18 score (global severity index, GSI) is a measure for general psychological distress. The German version of BSI-18 showed acceptable to good psychometric properties in several studies in Germany (Franke et al., 2017; Spitzer, 2011) and in a large representative sample in Switzerland (Michel et al., 2024). The GSI exhibited excellent internal consistency, with a Cronbach’s α of 0.91 at all waves.

### Mediator variables

*Perseverative Thinking* defined as content-independent repetitive negative thinking, was measured using the Perseverative Thinking Questionnaire (PTQ; (Ehring et al., 2011)). The PTQ comprises 15 items assessing features such as repetitiveness, intrusiveness, difficulty disengaging, and unproductiveness, for example, “I keep thinking about the same issue all the time”. Responses range from 0 = “never” to 4 = “almost always”. The German version of the PTQ showed high internal consistency, re-test reliability and concurrent validity (ibid). Internal consistency was excellent in the present study (Cronbach’s α = 0.95 at all waves).

*Emotional reactivity* was assessed using the Emotional Reactivity Scale (ERS; (Nock et al., 2008), a 21-item self-report instrument capturing context-independent sensitivity, intensity, and duration of emotional responses. Items such as “I tend to get emotional very easily” were rated on a 5-point scale (0 = “not at all like me” to 4 = “completely like me”). In the present sample, the scale demonstrated excellent internal consistency (Cronbach’s α = 0.91 at W1, 0.94 at W2 and W3). Satisfactory construct validity, high internal consistency, convergent validity, and measurement invariance for gender was confirmed in a Swiss sample of young adults (Lüönd et al., 2023). A recent study among emerging adults confirmed a one factor structure of the ERS and measurement invariance for gender (Ryder et al., 2025).

*Threat interpretation bias* was assessed with the Interpretation Bias Index for PTSD (IBIP; (Boffa et al., 2018). This 13-item task adapts the Word–Sentence Association Paradigm to assess automatic cognitive appraisals of trauma-relevant ambiguous scenarios, such as “A past memory suddenly comes to mind — scared”. Participants are presented with each scenario and a threatening interpretation, rating how strongly they associate the two on a scale from 0 = “not associated” at all to 6 = “strongly associated”. The IBIP has shown good discriminant validity and was able to predict PTSD diagnosis above and beyond a PTSD symptom checklist (ibid). IBIP scores were associated with PTSD symptom severity among trauma-exposed adults (Arditte Hall & Arditte, 2024). Consistent with previous research (Arditte Hall & Arditte, 2024; Boffa et al., 2018), in the present sample the IBIP demonstrated good internal consistency (Cronbach’s α between 0.88 and 0.89).

*Rejection sensitivity* was assessed as anxious expectations for rejection by significant others (e.g. friends, parents or partner) using the Rejection Sensitivity Questionnaire (RSQ, Berenson et al., 2009). The RSQ includes nine hypothetical interpersonal scenarios involving potential rejection. Participants rate the level of concern about the outcome of each situation (1 = “very unconcerned” to 6 = “very concerned”) and the perceived likelihood of rejection (1 = “very unlikely” to 6 = “very likely”). Composite scores were calculated by multiplying concern and likelihood ratings for each scenario and averaging across all items. An adequate factor structure and evidence for criterion validity for depression and anxiety was confirmed, however test-retest reliability in a subsample of participants was low (Mishra & Allen, 2025). The RSQ demonstrated good internal consistency in our sample (Cronbach’s α between 0.83 and 0.86.).

Sociodemographic variables included age, gender (1= male, 2 = female, 3 = non-binary), nationality (1 = Swiss, 2 = Swiss, at least one parent with migration background, 3 = non-Swiss nationality) and the country of origin of both parents, as well as the participant’s perceived financial burden at W1 and the perceived family financial burden during childhood (1 = “very easy to get by” to 6 = “very difficult to get by”).

## Statistical analysis

### Factor analysis of adverse childhood experiences (ACEs)

A factor analytic approach was employed to derive latent factors of the clustering of ACEs. This method allows for a more nuanced understanding of ACEs, as theoretically relevant measures are combined to derive empirically distinct factors, and factor loadings reflect the strength of the association between each indicator and the latent construct, with higher loadings indicating greater explanatory power in the outcomes (Brown, 2015). First, an Exploratory Factor Analysis (EFA) was conducted on a random split-half of the sample. Then, based on these findings, we conducted a Confirmatory Factor Analysis (CFA) on the second split-half sample. The factor structure was consistent across both halves and the full sample. Therefore, CFA results are reported for the full sample (*N* = 1934) to maximize power.

Due to estimation issues—specifically, non-positive definite matrices when including the full measurement model in mediation analyses—the saved factor scores were used in all subsequent mediation analyses (Asparouhov & Muthen, 2010). These empirically extracted and saved factors were entered as separate predictors in the mediation path models. A detailed description of the factor analyses and the results are provided in the Supplemental Material 1: Details factor analyses.

### Mediation models

As a first step, we estimated four separate longitudinal path models—one for each mediator—using a simplified version of the dual-process simplex model with lagged *b* paths and contemporaneous residual covariance paths proposed by Goldsmith et al. (2018), see Fig. 1b Each model controlled for previous levels of both the mediator and the outcome variable to minimize bias due to cross-sectional associations contaminating the estimation of *b* paths. Following Goldsmith et al., we set each variable’s autocorrelations over time to be equal, allowed for covariances between baseline mediator and outcome at W1 but constrained covariances between the mediator and outcome to be equal at the other the timepoints. Unstandardised bootstrapped confidence intervals for the total and the indirect effects were obtained using the model constraint command, but Mplus does not provide standardised coefficients for the overall indirect and the overall direct effects.

As a second step, all significant mediators were simultaneously included in a multiple mediation model to assess their relative contributions. To achieve an acceptable model fit, covariances between mediators at all timepoints were allowed. This design allows for the simultaneous evaluation of multiple, conceptually distinct and clinically relevant mediators within a multiple mediation framework, while accounting for temporal precedence and reducing potential bias due to cross-sectional mediation. By modelling these processes over time, we aimed to identify specific emotional and cognitive pathways through which ACEs exert their impact on later psychological functioning.

Descriptive analyses were conducted using Stata version 18 and the mediation analyses in Mplus version 8.11. Missing data were handled using the default full information maximum likelihood (FIML) estimation method in Mplus. Parameter estimates were bootstrapped with 5000 resamples to obtain robust standard errors and confidence intervals.

*Confounders* Preliminary analyses identified gender, nationality, perceived current financial burden and perceived family financial burden during childhood as correlates of all ACE factors and psychopathology. As only gender demonstrated significant associations (*p* < .01) with mediators and outcomes at W2 and W3, the models retained all confounders at W1, but only gender was included as a covariate at W2 and W3.

## Results

### Sample description

The mean age of participants at W1 was 19 years, and 66.8 % identified as women, see Table 1. A majority (68.3 %) held Swiss nationality, while 8.1 % were nationals of other European countries. Additionally, 4.3 % of participants had at least one parent with an Asian background, followed by 2.2 % with a Latin American, 1.5 % with an African or Middle Eastern, and 0.8 % with a North American (USA, Canada) or Australian background.

**Table 1:** Sample description at W1 (*N* = 1934).

	Mean / n	SD /%
**Age** (mean / SD)	19.40	1.33
**Gender**		
- male	604	31.4
- female	1286	66.8
- non-binary, other	35	1.8
**Relationship status**		
- single	1162	63.6
- in a relationship	644	35.2
- married	20	1.1
- divorced/separated	1	0.1
**Living situation**		
- living alone	69	3.8
- with parents	1499	81.6
- with partner	43	2.3
- with friends/colocation	162	8.8
- other	64	3.5
**Professional situation**		
- vocational training	417	22.7
- upper secondary education	434	23.7
- university	547	29.8
- employed	317	17.3
- unemployed	39	2.1
- gap year	80	4.4
**Nationality**		
- Swiss	1314	68.3
- Swiss, at least one parents with migration back ground	419	21.8
- Non-Swiss nationality	192	10.0
**Perceived current financial burden** (1–6, higher values indicate higher burden)	2.11	1.20
**Perceived family financial burden in childhood** (1–6, higher values indicate higher burden)	2.19	1.10
**Number of ACE types**		
- 0	425	21.5
- 1	526	26.6
- 2	352	17.8
- 3	255	12.9
- 4	193	9.8
- 5	139	7.0
- 6, 7	87	4.4
**Sum score CTQ and MACE items**	53.38	15.41
**Sum score of the BSI-18 (GSI)**	31.78	11.34

### Adverse childhood experiences

A total of 80 % of the participants reported at least one ACE, see Table 1. One ACE was reported by 26.6 % of the sample, 51.9 % reported at least two ACEs. Using the cut-offs defined in Häuser et al. (2011), the most frequent types of child maltreatment were emotional neglect (38.4 %) and emotional abuse (30.8 %). Prevalence for physical neglect was 11.4 %, 7.1 % for physical abuse and 17.5 % for sexual abuse. About 20 % of the participants reported “witnessing the parents arguing fiercely” and verbal peer victimization (“being called names”, “verbal abuse by peers” and “excluded by peers”) often or very often. For detailed prevalences see (Marmet et al., 2024).

*Factor analyses of ACEs*: To identify underlying distinct factors of the seven assessed ACE types, we employed a factor analytic approach, see Supplemental Material 1: Details factor analyses. The four-factor model of the CFA demonstrated good fit and identified three meaningful ACE domains which showed excellent reliability:

1) Family maltreatment, 18 items, including CTQ items for abuse and neglect as well as the MACE items for witnessing domestic violence (Cronbach’s α = 0.89, McDonald’s Ω = 0.96).
2) Verbal and physical peer victimization, all respective 5 MACE items (Cronbach’s α =0.86 McDonald’s Ω = 0.94), and
3) Sexual abuse, 6 items including all sexual abuse items of the respective CTQ scale and sexual abuse by peers (Cronbach’s α = 0.89, McDonald’s Ω = 0.97).

A fourth factor comprised all positively phrased and recoded items (i.e., all CTQ emotional neglect items and two CTQ physical neglect items: “Got taken care of” and “Got taken to the doctor when needed”). This indicates that the factor functioned as a method factor (Podsakoff et al., 2024). Because it was also strongly correlated with the family maltreatment factor (*r* = 0.80), it was excluded from the analyses, while still contributing to the estimation of the ACE factor model.

### Zero-order correlations

Table 2 presents the zero-order correlations of all variables included in the analyses. Family maltreatment, peer victimization and sexual abuse factors were each significantly correlated with psychopathology at all waves, as well as with all mediator variables across all time points. The effect sizes were generally in the moderate range (*r* = 0.23 - 0.49, except the correlation between sexual abuse and rejection sensitivity at W2 was *r* = 0.18).

**Table 2:** Zero-order correlations of adversity domains (1–3), psychopathology (4–6), mediators (7–18) and socio-demographic variables (19–22).

		Variable	1	2	3	4	5	6	7	8	9	10	11	12	13	14	15	16	17	18	19	20	21
ACE	1	Family maltreatment																					
	2	Sexual abuse	**.67**																				
	3	Peer victimization	**.51**	**.56**																			
BSI	4	BSI-18 W1	**.49**	**.45**	**.42**																		
	5	BSI-18 W2	**.41**	**.38**	**.37**	**.65**																	
	6	BSI-18 W3	**.37**	**.39**	**.36**	**.58**	**.70**																
Mediators	7	PTQ W1	**.36**	**.31**	**.31**	**.59**	**.43**	**.42**															
	8	PTQ W2	**.31**	**.25**	**.28**	**.47**	**.58**	**.52**	**.64**														
	9	PTQ W3	**.33**	**.25**	**.30**	**.42**	**.45**	**.59**	**.59**	**.67**													
	10	IBIPW1	**.43**	**.40**	**.41**	**.60**	**.49**	**.42**	**.55**	**.44**	**.38**												
	11	IBIPW2	**.41**	**.36**	**.37**	**.53**	**.58**	**.49**	**.46**	**.55**	**.42**	**.73**											
	12	IBIPW3	**.46**	**.39**	**.39**	**.52**	**.51**	**.58**	**.45**	**.49**	**.56**	**.69**	**.78**										
	13	ERS W1	**.32**	**.31**	**.34**	**.54**	**.46**	**.38**	**.64**	**.50**	**.47**	**.59**	**.50**	**.49**									
	14	ERS W2	**.27**	**.27**	**.32**	**.44**	**.54**	**.44**	**.49**	**.61**	**.50**	**.50**	**.56**	**.51**	**.79**								
	15	ERS W3	**.31**	**.29**	**.34**	**.44**	**.48**	**.53**	**.45**	**.54**	**.65**	**.47**	**.52**	**.59**	**.73**	**.81**							
	16	RSQ W1	**.31**	**.23**	**.28**	**.44**	**.33**	**.29**	**.44**	**.35**	**.33**	**.49**	**.43**	**.42**	**.41**	**.34**	**.33**						
	17	RSQ W2	**.25**	**.18**	**.27**	**.37**	**.37**	**.31**	**.35**	**.41**	**.35**	**.39**	**.49**	**.42**	**.32**	**.36**	**.34**	**.63**					
	18	RSQ W3	**.30**	**.25**	**.28**	**.40**	**.41**	**.46**	**.38**	**.41**	**.50**	**.40**	**.47**	**.51**	**.33**	**.36**	**.42**	**.62**	**.67**				
Sociodem.	19	Gender^a^	**.10**	**.20**	.04	**.18**	**.19**	**.14**	**.13**	**.14**	**.13**	**.16**	**.13**	**.11**	**.28**	**.28**	**.29**	.00	−0.02	.01			
	20	Nationality^b^	**.24**	**.17**	**.10**	**.16**	**.13**	**.10**	**.09**	.07	.06	**.09**	**.14**	**.10**	**.06**	.05	.03	**.08**	**.08**	.04	−0.02		
	21	Familiy financial burden childhood^c^	**.34**	**.23**	**.23**	**.21**	**.22**	**.19**	**.17**	**.18**	**.12**	**.21**	**.24**	**.25**	**.13**	**.13**	**.14**	**.18**	**.18**	**.19**	.01	**.16**	
	22	Participant financial burden^c^ W1	**.32**	**.23**	**.21**	**.28**	**.27**	**.27**	**.20**	**.18**	**.15**	**.25**	**.23**	**.26**	**.20**	**.18**	**.16**	**.19**	**.17**	**.17**	**.06**	**.10**	**.43**

Note: Significant correlations are bold; BSI-18 Brief Symptom Inventory; PTQ Perseverative Thinking Questionnaire; IBIP Interpretation Bias Index for PTSD (threat interpretation bias); ERS Emotional Reactivity Scale; RSQ Rejection Sensitivity Questionnaire.

a gender 1 = male, 2 = female; ^b^1 = Swiss Nationality, 2 = one parent with migration background, 3 = two parent with migration background; ^c^Perceived financial burden (1 = “very easy” to 6 = “very difficult”).

### Specific associations between domains of ACEs, psychopathology in emerging adulthood, and emotional processing and social information processing

Fig. 1b depicts the three-factor, dual-process simplex mediation model, incorporating lagged b-paths. Table 3 reports the direct path estimates for all four mediators, while Table 4 presents the corresponding indirect effects. All analyses were controlled for nationality, perceived current financial burden and perceived family financial burden during childhood and of the participants at W1 and gender at all waves.

**Table 3:** Longitudinal mediation models: direct paths from the adversity domains to psychopathology for the single mediation models*.*

Mediator	Adversity			Wave 1					Wave 2					Wave 3		
		path^b^	est.	95 % CI	p	std.	path^b^	est.	95 % CI	p	std.	path^b^	est.	95 % CI	p	std.
**PTQ**	P/E M	**a11**	**0.21**	**[0.14, 0.27]**	**<0.001**	**0.22**	**a12**	**0.08**	**[0.01, 0.14]**	**.022**	**0.08**	**a13**	**0.10**	**[0.01, 0.18]**	**.019**	**0.09**
	PV	**a21**	**0.15**	**[0.09, 0.20]**	**<0.001**	**0.16**	**a22**	**0.07**	**[0.01, 0.12]**	**.015**	**0.07**	**a23**	**0.08**	**[0.02, 0.14]**	**.009**	**0.08**
	SA	**a31**	0.05	[−0.03, 0.13]	.186	0.05	a32	−0.06	[−0.14, 0.02]	.152	−0.05	a33	−0.02	[−0.11, 0.07]	.621	−0.02
		**–**	**–**	**–**	**–**	**–**	**b1**	**0.05**	**[0.01, 0.09]**	**.011**	**0.07**	**b2**	**0.09**	**[0.05, 0.13]**	**<0.001**	**0.13**
**ERS**	P/E M	**a11**	**0.13**	**[0.07, 0.19]**	**<0.001**	**0.14**	a12	0.01	[−0.04, 0.06]	.757	0.01	**a13**	**0.06**	**[0.00, 0.13]**	**.036**	**0.06**
	PV	**a21**	**0.20**	**[0.16, 0.25]**	**<0.001**	**0.23**	**a22**	**0.06**	**[0.01, 0.10]**	**.012**	**0.06**	**a23**	**0.06**	**[0.01, 0.11]**	**.015**	**0.06**
	SA	a31	0.02	[−0.05, 0.10]	.556	0.02	a32	−0.03	[−0.10, 0.04]	.364	−0.03	a33	−0.01	[−0.08, 0.06]	.842	−0.01
		**–**	**–**	**–**	**–**	**–**	**b1**	**0.08**	**[0.05, 0.12]**	**<0.001**	**0.11**	b2	0.03	[−0.01, 0.08]	.124	0.11
**IBIP**	P/E M	**a11**	**0.28**	**[0.21, 0.36]**	**<0.001**	**0.22**	**a12**	**0.10**	**[0.01, 0.19]**	**.025**	**0.08**	**a13**	0.19	**[0.09, 0.28]**	**<0.001**	**0.14**
	PV	**a21**	**0.27**	**[0.21, 0.33]**	**<0.001**	**0.22**	**a22**	**0.08**	**[0.01, 0.16]**	**.024**	**0.06**	a23	0.07	[−0.01, 0.14]	.066	0.05
	SA	**a31**	**0.13**	**[0.04, 0.24]**	**.009**	**0.09**	a32	−0.01	[−0.11, 0.10]	.923	0.00	a33	0.05	[−0.05, 0.16]	.324	0.03
		**–**	**–**	**–**	**–**	**–**	**b1**	**0.07**	**[0.04, 0.10]**	**<0.001**	**0.13**	**b2**	**0.04**	**[0.00, 0.08]**	**.041**	**0.08**
**RSQ**	P/E M	**a11**	**0.96**	**[0.64, 1.29]**	**<0.001**	**0.20**	a12	0.17	[−0.17, 0.48]	.323	0.04	a13	0.39	[−0.04, 0.79]	.071	0.08
	PV	**a21**	**0.77**	**[0.50, 1.03]**	**<0.001**	**0.17**	**a22**	**0.47**	**[0.16, 0.79]**	**.003**	**0.10**	a23	0.14	[−0.17, 0.46]	.378	0.03
	SA	a31	−0.14	[−0.55, 0.29]	.500	−0.03	a32	−0.25	[−0.67, 0.21]	.266	−0.04	a33	0.45	[−0.10, 1.01]	.115	0.08
		–	–	–	–	–	b1	0.01	[0.00, 0.01]	.146	0.04	b2	0.01	[0.00, 0.01]	.146	0.04
**BSI**^a^	P/E M	**c’11**	**0.19**	**[0.14, 0.23]**	**<0.001**	**0.26**	**c’12**	**0.05**	**[0.01, 0.10]**	**.033**	**0.08**	c’13	0.00	[−0.05, 0.05]	.919	0.00
	PV	**c’21**	**0.14**	**[0.10, 0.18]**	**<0.001**	**0.20**	**c’22**	**0.06**	**[0.02, 0.10]**	**.002**	**0.09**	c’23	0.03	[−0.01, 0.07]	.139	0.05
	SA	**c’31**	**0.10**	**[0.04, 0.16]**	**.001**	**0.11**	c’32	0.01	[−0.05, 0.07]	.654	0.02	**c’33**	**0.09**	**[0.03, 0.16]**	**.003**	**0.11**

Note: Significant results are shown in bold; results are controlled for the confounders. est.: unstandardised estimate; std.: standardised estimate; PTQ: Perseverative Thinking Questionnaire; IBIP: Interpretation Bias for Threat Relevance Questionnaire; ERS: Emotional Reactivity Scale; RSQ: Rejection Sensitivity Questionnaire; BSI-18: Brief Symptom Inventory.

a Results for the associations between adversity domains and the BSI-18 based on the model with the PTQ as mediator. The results are in the same range as in the models with the other mediators.

b Paths are described in Fig. 1ba paths represents the paths from the adversity domains to psychopathology (x–>*Y*); b1 represents the lagged path from the putative mediator at Wave 1 to subsequent psychopathology at Wave 2 (M1–> Y2), b2 represents the lagged path from the putative mediator at Wave 2 to subsequent psychopathology at Wave 3 (M2–> Y3); c’ paths represent the paths from the adversity domains to psychopathology controlling for the mediator.

**Table 4:** Longitudinal mediation models: indirect effects of single mediators on psychopathology at wave 3.

Mediator	Family maltreatment				Peer victimization				Sexual abuse			
	est	95 % CI	p	std	est	95 % CI	p	std	est	95 % CI	p	std
Perseverative thinking												
Overall indirect effect	**0.02**	**[0.01, 0.04]**	**<0.001**	**n/a**	**0.02**	**[0.01, 0.03]**	**<0.001**	**n/a**	0.00	[−0.01, 0.01]	.834	n/a
x->M1 ->Y2->Y3	**0.01**	**[0.00, 0.01]**	**.024**	**0.01**	**0.00**	**[0.00, 0.01]**	**.030**	**0.01**	0.00	[0.00, 0.00]	.277	0.00
x->M1 ->M2->Y3	**0.01**	**[0.01, 0.02]**	**.001**	**0.02**	**0.01**	**[0.00, 0.01]**	**.001**	**0.01**	0.00	[0.00, 0.01]	.225	0.00
x->M2->Y3	0.01	[0.00, 0.02]	.062	0.01	**0.01**	**[0.00, 0.01]**	**.035**	**0.01**	−0.01	[−0.01, 0.00]	.191	−0.01
Overall direct effect	**0.08**	**[0.02, 0.13]**	**.010**	**n/a**	**0.10**	**[0.05, 0.14]**	**<0.001**	**n/a**	**0.12**	**[0.05, 0.20]**	**.001**	**n/a**
x->Y1 -> Y2->Y3	**0.04**	**[0.03, 0.06]**	**<0.001**	**0.07**	**0.03**	**[0.02, 0.04]**	**<0.001**	**0.05**	**0.02**	**[0.01, 0.04]**	**.002**	**0.03**
x-> Y2->Y3	**0.03**	**[0.00, 0.06]**	**.033**	**0.04**	**0.03**	**[0.01, 0.05]**	**.003**	**0.05**	0.01	[−0.02, 0.04]	.656	0.01
x->Y3	0.00	[−0.05, 0.05]	.919	0.00	0.03	[−0.01, 0.07]	.139	0.05	**0.09**	**[0.03, 0.16]**	**.003**	**0.11**
Total effect	**0.10**	**[0.04, 0.15]**	**.001**	**0.15**	**0.11**	**[0.07, 0.16]**	**<0.001**	**0.17**	**0.12**	**[0.05, 0.20]**	**.002**	**0.15**
% total by indirect	24.49				15.93				0.82			
Emotional reactivity												
Overall indirect effect	**0.01**	**[0.00, 0.02]**	**.006**	**n/a**	**0.02**	**[0.01, 0.03]**	**<0.001**	**n/a**	0.00	[−0.01, 0.01]	.840	n/a
x->M1 ->Y2->Y3	**0.01**	**[0.00, 0.01]**	**.004**	**0.01**	**0.01**	**[0.01, 0.02]**	**<0.001**	**0.02**	0.00	[0.00, 0.01]	.571	0.00
x->M1 ->M2->Y3	0.00	[0.00, 0.01]	.151	0.01	0.01	[0.00, 0.01]	.131	0.01	0.00	[0.00, 0.00]	.632	0.00
x->M2->Y3	0.00	[0.00, 0.00]	.795	0.00	0.00	[0.00, 0.01]	.222	0.00	0.00	[0.00, 0.00]	.498	0.00
Overall direct effect	**0.09**	**[0.04, 0.15]**	**.001**	**n/a**	**0.10**	**[0.05, 0.14]**	**<0.001**	**n/a**	**0.12**	**[0.04, 0.20]**	**.002**	**n/a**
x->Y1 -> Y2->Y3	**0.05**	**[0.04, 0.07]**	**<0.001**	**0.07**	**0.04**	**[0.03, 0.05]**	**<0.001**	**0.06**	**0.03**	**[0.01, 0.04]**	**.001**	**0.03**
x-> Y2->Y3	0.03	[0.00, 0.06]	.036	0.04	**0.03**	**[0.01, 0.05]**	**.012**	**0.04**	0.01	[−0.03, 0.04]	.757	0.01
x->Y3	**0.01**	**[−0.04, 0.06]**	.588	0.02	**0.03**	[−0.01, 0.07]	.139	0.05	**0.09**	**[0.03, 0.15]**	**.006**	**0.10**
Total effect	**0.10**	**[0.05, 0.16]**	**<0.001**	**0.15**	**0.11**	**[0.07, 0.16]**	**<0.001**	**0.18**	**0.12**	**[0.04, 0.20]**	**.002**	**0.14**
% total by indirect	**8.82**				**15.18**				0.85			
Threat interpretation bias												
Overall indirect effect	**0.02**	**[0.01, 0.04]**	**.001**	**n/a**	**0.02**	**[0.01, 0.03]**	**.001**	**n/a**	0.01	[0.00, 0.02]	.051	n/a
x->M1 ->Y2->Y3	**0.01**	**[0.01, 0.02]**	**.001**	**0.02**	**0.01**	**[0.01, 0.02]**	**.001**	**0.02**	**0.01**	**[0.00, 0.01]**	**.035**	**0.01**
x->M1 ->M2->Y3	**0.01**	**[0.00, 0.02]**	**.049**	**0.01**	**0.01**	**[0.00, 0.02]**	**.050**	**0.01**	0.00	[0.00, 0.01]	.122	0.00
x->M2->Y3	0.00	[0.00, 0.01]	.134	0.01	0.00	[0.00, 0.01]	.165	0.01	0.00	[−0.01, 0.01]	.930	0.00
Overall direct effect	**0.08**	**[0.03, 0.14]**	**.007**	**n/a**	**0.09**	**[0.05, 0.14]**	**<0.001**	**n/a**	**0.11**	**[0.05, 0.19]**	**.005**	**n/a**
x->Y1 -> Y2->Y3	**0.04**	**[0.03, 0.06]**	**.000**	**0.07**	**0.03**	**[0.02, 0.04]**	**<0.001**	**0.05**	**0.02**	**[0.01, 0.04]**	**.002**	**0.03**
x-> Y2->Y3	**0.03**	**[0.00, 0.06]**	**.039**	**0.04**	**0.03**	**[0.01, 0.05]**	**.008**	**0.05**	0.00	[−0.03, 0.03]	.938	0.00
x->Y3	0.01	[−0.04, 0.06]	.760	0.01	0.03	[−0.01, 0.07]	.168	0.05	**0.08**	**[0.03, 0.15]**	**.008**	**0.10**
Total effect	0.10	[0.06, 0.16]	**<0.001**	0.15	**0.11**	**[0.07, 0.16]**	**<0.001**	**0.17**	**0.12**	**[0.05, 0.20]**	**.003**	**0.14**
% total by indirect	**21.78**				**18.92**				**6.90**			
Rejection sensitivity												
Overall indirect effect	0.01	[0.00, 0.02]	.105	n/a	0.01	[0.00, 0.02]	.123	n/a	0.00	[−0.01, 0.00]	.414	n/a
x->M1 ->Y2->Y3	0.00	[0.00, 0.01]	.162	0.01	0.00	[0.00, 0.01]	.155	0.00	0.00	[0.00, 0.00]	.602	0.00
x->M1 ->M2->Y3	0.00	[0.00, 0.01]	.276	0.01	0.00	[0.00, 0.01]	.272	0.00	0.00	[0.00, 0.00]	.657	0.00
x->M2->Y3	0.00	[0.00, 0.00]	.533	0.00	0.00	[0.00, 0.01]	.308	0.00	0.00	[−0.01, 0.00]	.498	0.00
Overall direct effect	**0.10**	**[0.04, 0.15]**	**.001**	**n/a**	**0.10**	**[0.06, 0.15]**	**<0.001**	**n/a**	**0.12**	**[0.04, 0.20]**	**.003**	**n/a**
x->Y1 -> Y2->Y3	**0.05**	**[0.04, 0.07]**	**.000**	**0.08**	**0.04**	**[0.03, 0.05]**	**<0.001**	**0.06**	**0.03**	**[0.01, 0.04]**	**.002**	**0.03**
x-> Y2->Y3	**0.03**	**[0.00, 0.06]**	**.032**	**0.05**	**0.03**	**[0.01, 0.06]**	**.003**	**0.05**	0.01	[−0.03, 0.04]	.737	0.01
x->Y3	0.02	[−0.03, 0.07]	.481	0.03	0.03	[−0.01, 0.08]	.095	0.06	**0.08**	**[0.03, 0.15]**	**.007**	**0.11**
Total effect	**0.10**	**[0.05, 0.16]**	**<0.001**	**0.16**	**0.11**	**[0.06, 0.16]**	**<0.001**	**0.18**	**0.12**	**[0.04, 0.20]**	**.004**	**0.14**
% total by indirect	6.73				6.25				1.74			

Note: Significant results are shown in bold. Results are controlled for the confounders. X: Adversity domain; M1: Mediator at Wave 1; M2: Mediator at Wave 2; Y1: Psychopathology (BSI-18) at Wave 1; Y2: Psychopathology (BSI-18) at Wave 2; Y3: Psychopathology (BSI-18) at Wave 3. PTQ: Perseverative Thinking Questionnaire; IBIP: Interpretation Bias Index for PTSD; ERS: Emotional Reactivity Scale; est.: unstandardised estimate; std.: standardised estimate.

*Specific associations of domains of ACEs with psychopathology:* All three maltreatment factors—family maltreatment, sexual abuse, and peer victimization—were significantly associated with higher psychopathology at W1. At W2, controlling for psychopathology levels at W1, *family maltreatment* and *peer victimization* remained significantly associated with psychopathology at W3, whereas *sexual abuse* did not. Conversely, only *sexual abuse* directly predicted additional psychopathology at W3. These findings suggest that each domain of maltreatment not only contributed to initial levels of psychopathology but also had a continued, additive effect on future psychopathology beyond what can be explained by prior symptom levels.

*Specific associations of domains of ACEs with emotion processing and social information processing variables at W1:* As hypothesized, family maltreatment and peer victimization were each significantly associated with higher emotional reactivity, perseverative thinking, rejection sensitivity, and threat interpretation bias at W1. In contrast to our hypotheses, *sexual abuse* was only significantly associated with threat interpretation bias at W1 but no other mediators at W1, W2 or W3.

*Specific continued effects of domains of ACEs with emotional processing and social information processing variables at W2 and W3: Family maltreatment* showed continued effects (i.e. remaining direct effect after controlling for autoregression) with perseverative thinking and threat interpretation bias at W2 and W3, continued effects with emotional reactivity at W3 but not with rejection sensitivity at W2 or W3, controlling for each mediator's level at the previous waves. *Peer victimization* showed continued effects with perseverative thinking and emotional reactivity across all waves. Furthermore, peer victimization predicted threat interpretation bias and rejection sensitivity at W2 but not W3. These findings suggest that *family maltreatment and peer victimization*, in contrast to *sexual abuse,* had continued, additive effects on emotion processing and social information processing variables beyond what can be explained by prior levels.

### Lagged effects of emotion processing and social information processing variables on subsequent psychopathology

In line with our hypotheses, elevated levels of *perseverative thinking* and *threat interpretation* bias at W1 and W2 significantly predicted higher psychopathology at the subsequent waves. Higher *emotional reactivity* predicted increased psychopathology from W1 to W2 but not from W2 to W3, when controlling for prior levels of emotional reactivity. In contrast to our hypotheses, higher levels of *rejection sensitivity* did not predict higher subsequent psychopathology either at W2 or W3, suggesting no pathway for indirect effects from ACEs to psychopathology through this social information processing variable. Consequently, the hypothesis that rejection sensitivity mediates the relationship between domains of ACEs and later psychopathology was not supported.

### Mediators of ACEs on psychopathology at waves 2 and 3

For the total indirect effects, we calculated the sum of X→M1→Y2→Y3 (proximal effect), X→M1→ M2→Y3 (sustained effect) and X→M2→Y3 (distal effect). Total effects from *family maltreatment* and *peer victimization* to subsequent psychopathology at W2 and W3 were mediated by emotional reactivity, perseverative thinking, and threat interpretation bias, as each exhibited small but statistically significant indirect effects. For *sexual abuse*, only threat interpretation bias functioned as a significant mediator, also with a small effect. Table 4 and Supplemental Material 2 detail the proximal, sustained and distal indirect effects exhibited by these mediators.

*Mediator explained variance at W3:* Each of the three significant mediators explained an equivalent amount of the total effects from peer victimization to W3 psychopathology (approximately a sixth of total effects, 15–19 %). However, from family maltreatment, more W3 effects were explained by threat interpretation bias and perseverative thinking (approximately a quarter, 22–25 %) than emotional reactivity (9 %). From sexual abuse, only 7 % of total effects were explained via threat interpretation bias.

## Multiple mediation model

In the next step, a multiple mediation model was tested, including the three significant mediators—perseverative thinking, emotional reactivity, and threat interpretation bias—to evaluate their relative independent contributions to subsequent psychopathology. Across both simple and multiple mediation models, the proportion of explained variance in psychopathology by the whole model was 34–35 % at W1, 40–42 % at W2, 46–49 % at W3.

*Lagged effects:* Only threat interpretation bias at W1 showed a unique and significant lagged effect on higher psychopathology at W2 (*B* = 0.04, 95 % *CI* [0.00, 0.07], *p* = .028, *beta* = 0.07), with perseverative thinking and emotional reactivity showing non-significant lagged effects. Then, only perseverative thinking at W2 predicted increased psychopathology at W3 (*B* = 0.07, 95 % *CI* [0.03, 0.12], *p* = .001, *beta* = 0.11). Notably, emotional reactivity at W2 had a small but significant effect for lower subsequent psychopathology (*B* = −0.05, 95 % *CI* [0.10, −0.01], *p* = .020, *beta* = −0.07). Lagged effects of W2 threat interpretation bias on W3 psychopathology were non-significant. Details of the proximal, sustained and distal indirect effects of the mediators on psychopathology at W2 and W3 are found in Table 5 and Supplemental Material 2: Details mediation analyses.

**Table 5:** Multiple longitudinal mediation model: indirect effects on psychopathology at wave 3.

Effects		Family maltreatment				Peer victimization				Sexual abuse		
	est	95 % CI	p	std	est	95 % CI	p	std	est	95 % CI	p	std
Overall indirect effect	**0.03**	**[0.01, 0.04]**	**<0.001**	**n/a**	**0.02**	**[0.00, 0.03]**	**.012**	**n/a**	0.00	[−0.01, 0.01]	.480	n/a
x ->M1_ptq_ ->Y2->Y3	0.00	[0.00, 0.01]	.400	0.00	0.00	[0.00, 0.01]	.405	0.00	0.00	[0.00, 0.00]	.542	0.00
x ->M1_ptq_ ->M2_ptq_ ->Y3	**0.01**	**[0.00, 0.02]**	**.004**	**0.01**	**0.01**	**[0.00, 0.01]**	**.007**	**0.01**	0.00	[0.00, 0.01]	.225	0.00
x ->M2_ptq_ ->Y3	0.01	[0.00, 0.01]	.074	0.01	**0.01**	**[0.00, 0.01]**	**.035**	**0.01**	−0.01	[−0.01, 0.00]	.175	−0.01
x ->M1_IBIP_ ->Y2->Y3	**0.01**	**[0.00, 0.01]**	**.042**	**0.01**	**0.01**	**[0.00, 0.01]**	**.042**	**0.00**	0.00	[0.00, 0.01]	.110	0.00
x ->M1_IBIP_ ->M2_IBIP_ ->Y3	0.00	[0.00, 0.01]	.242	0.01	0.00	[0.00, 0.01]	.240	0.01	0.00	[0.00, 0.01]	.305	0.00
x->M2_IBIP_ ->Y3	0.00	[0.00, 0.01]	.299	0.00	0.00	[0.00, 0.01]	.335	0.00	0.00	[0.00, 0.00]	.970	0.00
x->M1_ers_ ->Y2->Y3	0.00	[0.00, 0.01]	.284	0.00	0.00	[0.00, 0.01]	.269	0.00	0.00	[0.00, 0.00]	.685	0.00
x->M1_ers_ ->M2_ers_ ->Y3	**−0.01**	**[−0.01, 0.00]**	**.045**	**−0.01**	**−0.01**	**[−0.02, 0.00]**	**.030**	**−0.01**	0.00	[0.00, 0.00]	.607	0.00
x ->M2_ers_ ->Y3	0.00	[0.00, 0.00]	.734	0.00	0.00	[−0.01, 0.00]	.070	−0.01	0.00	[0.00, 0.01]	.468	0.00
Overall direct effect	**0.08**	**[0.02, 0.13]**	**.010**	**n/a**	**0.10**	**[0.05, 0.14]**	**<0.001**	**n/a**	**0.12**	**[0.04, 0.19]**	**.003**	**n/a**
x ->Y1 -> Y2->Y3	**0.04**	**[0.03, 0.05]**	**<0.001**	**0.06**	**0.03**	**[0.02, 0.04]**	**<0.001**	**0.05**	**0.02**	**[0.01, 0.04]**	**.002**	**0.03**
x -> Y2->Y3	**0.03**	**[0.00, 0.06]**	**.026**	**0.04**	**0.03**	**[0.01, 0.05]**	**.005**	**0.05**	**0.01**	[−0.03, 0.04]	.757	0.01
x ->Y3	0.01	[−0.04, 0.06]	.776	0.01	0.04	[−0.01, 0.08]	.081	0.06	**0.09**	**[0.03, 0.15]**	**.005**	**0.11**
Total effect	**0.10**	**[0.05, 0.16]**	**<0.001**	**0.22**	**0.11**	**[0.06, 0.16]**	**<0.001**	**0.20**	**0.12**	**[0.04, 0.20]**	**.003**	**0.07**
% total by indirect	**24.75**				**13.51**				3.36			

Note: Significant results are shown in bold. Results are controlled for confounders. X: Adversity domain; M1: Mediator at Wave 1; M2: Mediator at Wave 2; Y1: Psychopathology (BSI-18) at Wave 1; Y2: Psychopathology (BSI-18) at Wave 2; Y3: Psychopathology (BSI-18) at Wave 3. PTQ: Perseverative Thinking Questionnaire; IBIP: Interpretation Bias Index for PTSD; ERS: Emotional Reactivity Scale. est.: unstandardised estimate; std.: standardised estimate.

*Indirect effects:* Independent indirect effects were confirmed for family maltreatment and peer victimization, but in contrast to the single mediator model, not for sexual abuse. Consistent with the single mediator models, the relationships between *family maltreatment* and *peer victimization* and subsequent psychopathology at W3 were mediated by sustained indirect effects of perseverative thinking (X→M1→M2→Y3) and proximal indirect effects of threat interpretation bias (X→M1→Y2→Y3). Additionally, a distal indirect effect of perseverative thinking (X→M2→Y3) was confirmed for *peer victimization*.

In contrast to the single mediation models, the proximal indirect effects of perseverative thinking and the sustained indirect effects of threat interpretation bias were not supported. In line with the lagged effect model, a sustained indirect effect of emotional reactivity in the inverse direction was found for both *family maltreatment* and *peer victimization*; however, the proximal effect was not observed.

## Discussion

The present study investigated mechanisms mediating the link between domains of adverse childhood experiences (ACEs) and psychopathology in a large general population sample of emerging adults. In contrast to many prior studies, we assessed a comprehensive range of ACEs and employed a factor-analytic approach to identify distinct ACE domains of family maltreatment (including physical and emotional maltreatment and witnessing domestic violence), sexual abuse, and peer victimization. By modelling the ACE factors simultaneously, we minimised confounding by their frequent co-occurrence and isolated their unique contributions to later psychopathology.

Overall, our results showed that the effects of family maltreatment, peer victimization and sexual abuse on later psychopathology were partially mediated by emotion processing and social information processing components. Specifically, perseverative thinking, threat interpretation bias, and emotional reactivity emerged as robust mediators. Contrary to hypothesis, rejection sensitivity did not mediate the link between ACE and psychopathology. While family maltreatment and peer victimization showed similar associations with psychopathology and acted through similar pathways, sexual abuse showed weaker or delayed associations with psychopathology, with only threat interpretation bias emerging as a significant pathway.

### Associations of adversity domains with psychopathology, emotional processing and social information processing in emerging adulthood

*ACE domains predicting psychopathology in emerging adulthood:* Beyond the significant bivariate correlations between all adversity domains and psychopathology, all domains—family maltreatment, peer victimization, and sexual abuse, and—were found to independently predict higher subsequent psychopathology, even when controlling for all adversity domains, gender, nationality, perceived family financial burden during childhood, and participants’ current perceived financial burden at Wave 1. Thus, our findings confirmed the unique roles of multiple ACE domains in predicting psychopathology and extend previous research using a single-risk approach to child maltreatment (Guiney et al., 2024; Hashim et al., 2024, 2025; Kim & Royle, 2025; Kuzminskaite et al., 2021; Lee et al., 2025; Li et al., 2024; Rijlaarsdam et al., 2021; Trompeter et al., 2024).

*ACE domains and emotional processing and social information processing:* In unadjusted bivariate analyses, all three ACE factors were moderately correlated with the four potential mediators—emotional reactivity, perseverative thinking, rejection sensitivity, and threat interpretation bias. This is consistent with previous research (Gao et al., 2024; Lavi et al., 2019; Mansueto et al., 2021; Miu et al., 2022; Potter et al., 2025).

In adjusted multivariate longitudinal models, differences emerged that underscore the specificity of ACE domains in relation to emotional and social information processing. Family maltreatment and peer victimization each independently showed early and sustained effects on difficulties in all emotional processing and social information processing components, except the association of family maltreatment and rejection sensitivity was not sustained. In contrast, sexual abuse was independently associated only with threat interpretation bias at Wave 1, suggesting that the other putative mediators of emotional and social information processing are not relevant for this type of ACEs. These findings are in line with previous studies that found that sexual abuse was not independently associated with emotional reactivity (Ryder et al., 2025) or rejection sensitivity (Euteneuer et al., 2024), and was inconsistently associated with rumination (Mansueto et al., 2021).

*Prolonged Effects Across Time:* Furthermore, we found prolonged effects of ACE domains and the possible mediators, and psychopathology over time. Family maltreatment, as well as peer victimization, predicted not only concurrent but also persistent elevations in psychopathology, emotional dysregulation, and difficulties in social information processing. In contrast, sexual abuse did not predict psychopathology at Wave 2 but was the only dimension to show a prolonged effect on psychopathology at Wave 3. These findings suggest that, in this general population sample, there is little evidence for natural resilience processes at the group level. The typical trajectory does not appear to involve a gradual remission of symptoms or improvement in adaptive coping over time, but rather indicates continued or even escalating symptoms, alongside persistent emotional and cognitive dysregulation. This is consistent with the high increase in onset of psychological disorders between ages 18 to 25 (Solmi et al., 2022), highlighting the critical importance of early identification and timely intervention for distressed emerging adults with a history of ACEs.

### Emotional Processing and social information processing as mediators of the link between ACEs and psychopathology in emerging adulthood

Perseverative thinking, threat interpretation bias, and emotional reactivity—but not rejection sensitivity—were confirmed as mediators of the association between family maltreatment and peer victimization and later psychopathology. This finding aligns with, and extends, the Transdiagnostic Model of Mechanisms Linking Childhood Trauma to Psychopathology (McLaughlin et al., 2020) by underlining the importance of differentiating distinct threat-related adversity factors for the analysis of mechanisms. Also extending existing knowledge, the present findings offer a more nuanced view of the temporal dynamics of emotional and cognitive mediators in the pathway from distinct adversity domains to subsequent psychopathology.

*Perseverative thinking* (e.g., rumination and worry) was confirmed as a mediator of family maltreatment and peer victimization on psychopathology in single and multiple mediator models. Specifically, perseverative thinking showed both proximal effects (X→M1→Y2→Y3) and sustained indirect effects (X→M1→M2→Y3) on psychopathology at W3, with an additional distal indirect effect (X→M2→Y3) from peer victimization to psychopathology. These sustained effects support transdiagnostic models that conceptualize repetitive negative thinking as a stable, trait-like vulnerability that both amplifies distress in the short term and perpetuates it over time (Ehring & Watkins, 2008; Moulds & McEvoy, 2025). The emergence of an additional distal effect in the context of peer victimization may reflect re-traumatisation or the ongoing nature of perseverative thinking wherein negative thought processes are repeatedly activated by interpersonal stressors in emerging adulthood (McLaughlin & Nolen-Hoeksema, 2011). Taken together, these results suggest that the influence of perseverative thinking may intensify over time, thereby sustaining or even exacerbating psychopathological symptoms.

Emotional reactivity, in contrast to perseverative thinking, operated as a purely proximal mediator linking family maltreatment and peer victimization to psychopathology. This implies that heightened reactivity is not a stable trait-like vulnerability but one that may diminish over time by developing regulatory skills or supportive environments. Notably, when modelled alongside other mediators, greater emotional reactivity at Wave 2 predicted lower psychopathology at Wave 3. An interpretation might be that, after accounting for maladaptive cognitive processes, this residual variance reflects adaptive facets of emotional responsiveness that could facilitate coping or social adaptation.

The present study provides the first population based longitudinal evidence that *threat interpretation bias* mediates the association between all adversity domains and later psychopathology. This extends prior work, primarily using cross sectional designs with laboratory-based paradigms (Minihan et al., 2023; Weissman et al., 2019). In contrast to perseverative thinking, the effects of threat interpretation bias were more time limited, showing proximal and only marginally sustained effects for maltreatment and peer victimization, and a solely proximal effect for sexual abuse. Within the multiple mediator model, only the proximal pathways for maltreatment and peer victimization remained significant. These findings suggest that threat interpretation biases are activated after adversity and may precipitate symptom development but are less likely to persist autonomously once other mechanisms (e.g., perseverative thinking) gain prominence. We also note that our measure for threat interpretation bias, the Interpretation Bias Index for PTSD (Boffa et al., 2018), originally designed for tracking threat interpretation biases and screening for post-traumatic stress disorder in clinical samples, predicted transdiagnostic psychopathology in this population sample. This underscores its value as a brief self-report tool for large scale epidemiological research.

*Rejection sensitivity* did not emerge as a significant mediator in our results, in contrast to findings from Minihan et al. (2023). This divergence may stem from methodological differences: Minihan et al. (2023) analysed perceived parental rejection as ACE which may be more closely related to social rejection as mediator than our broader ACE domain of family maltreatment. Also, Minihan used an experimental paradigm, yet laboratory tasks might overestimate effects under controlled conditions. While our scenario based self-report measure RSQ has shown criterion validity for depression and anxiety (Mishra & Allen, 2025), it could nonetheless lack sensitivity. Future work should incorporate experience sampling designs to capture rejection sensitive reactions immediately following real world social interactions.

## Limitations

This longitudinal three‑wave study has several strengths, including a large sample of Swiss emerging adults recruited primarily through a random‑sampling approach. However, the relatively low response rate of 16 %, the overrepresentation of women and the Swiss context limit the generalizability of the findings.

Another limitation concerns the exclusive use of self-report questionnaires to assess all variables. Psychopathology was measured with the self-report BSI-18 and not confirmed with clinical diagnoses. Self-report measures are susceptible to recall and reporting biases, particularly in the retrospective assessment of ACEs (Hardt & Rutter, 2004). However, both prospective and retrospective methods may yield under- or over-reporting (Baldwin et al., 2019, 2024). Furthermore, our research question required a measurement model that distinguishes qualitatively different domains of co-occurring interpersonal adversity. Therefore, we used a reflective latent variable approach in contrast to a formative measurement model which would be appropriate to operationalize theoretically derived higher-order dimensions of ACEs—such as deprivation or threat—which are linked by their cumulative influence on outcomes. Additionally, the factor-analytic approach of ACE did not yield a distinct neglect factor. While the Childhood Trauma Questionnaire (CTQ) is widely used, its positively worded items for emotional neglect may have introduced methodological artefacts. This is reflected in the factor analysis, where emotional-neglect items (and two positively phrased physical-neglect items) loaded onto a factor that correlated highly with the broader family maltreatment factor. An alternative measure of emotional neglect or a diagnostic interview might have yielded clearer differentiation between abuse and neglect dimensions.

Furthermore, annual follow-ups may be too coarse to detect rapid developmental shifts and may have overlooked shorter-term fluctuations in the mediating processes. More frequent assessments or intensive longitudinal methods (e.g., monthly surveys, experience sampling) could capture dynamic mediation. While our models were temporally ordered, reverse or reciprocal effects (e.g., psychopathology predicting changes in mediators) were not tested. The temporal offset of variables in the mediation models and adjustment by confounds does not rule out residual confounding. Also, due to power issues the timing of the ACEs in childhood and adolescence could not be included as a moderator in the analyses. This may have an influence on the development of emotion processing and social information processing as well as the timing of effects observed in our models. Finally, the study was not sufficiently powered to adjust for multiple comparisons across the entire mediation model, increasing the risk of Type I error. Replication in larger samples is therefore essential.

## Theoretical and clinical implications

Our findings largely support emotional processing and social‑information processing as key mechanisms linking ACEs to psychopathology, as outlined in the Transdiagnostic Model of Mechanisms Linking Childhood Trauma to Psychopathology (McLaughlin et al., 2020). Our study extends the model by differentiating specific threat-related adversity domains, i.e. family maltreatment, peer victimisation and sexual abuse. Our findings underscore that different ACEs partially operate through distinct pathways and therefore highlight the importance of disaggregating ACE types to capture nuanced aetiological routes to psychopathology. *Peer victimization* and *family maltreatment*—in contrast to *sexual abuse*—displayed comparable associations with psychopathology and shared the same three mediators— emotional reactivity, perseverative thinking, and threat interpretation bias. This supports the call that peer victimization should be integrated into the ACE framework (Karatekin & Hill, 2019)*.* Nevertheless, as peer-relationships tend to be more central in emerging adulthood than in older age (Arnett, 2015), further studies should test whether these associations with psychopathology persist into later adulthood and in general whether ACE effects on psychopathology are sustained into later adulthood by the same processes.

Our study underscores the importance of integrated, early, and scalable interventions tailored to specific ACE domains and their psychological mechanisms. About 80 % of the sample reported at least one ACE and, at the population level, the natural course after experiencing ACEs is towards persistence or worsening of emotional and social information processing, and psychopathology. Thus, emerging adulthood seems a vulnerable period for individuals with a history of ACEs. Early transdiagnostic interventions or secondary prevention programs targeting threat interpretation bias, perseverative thinking and emotional reactivity may address these issues. Cognitive restructuring that challenges threat interpretation biases and reframes social threat perceptions may be particularly beneficial. This is crucial since such biases served as unique mediators—even for sexual abuse.

Furthermore, for individuals with pronounced psychopathological distress, the differential associations of ACE domains with mediators suggest the relevance of personalized and mechanism-based treatment planning based on adversity profiles. For example, in individuals with a history of family maltreatment or peer victimisation, targeting perseverative thinking and threat interpretation biases appears more promising than focusing on emotional reactivity. Emerging adults with a history of sexual abuse may benefit from different or supplementary therapeutic approaches.

## Further research

Further research should investigate protective factors and resilience processes as potential mediators such as social support as a buffering factor (McLaughlin et al., 2020). Additionally, research with more waves of data could focus on the interplay among emotional reactivity, rejection sensitivity, perseverative thinking, and threat interpretation bias. Serial mediation models may be employed to examine how these processes mediate the relationship between specific domains of adverse childhood experiences (ACEs)—ideally recorded prospectively—and later psychopathology. As the BSI-18 primarily captures internalizing symptoms, future studies should also explore whether externalizing problems and psychotic disorders follow different patterns of association and mediation. Moreover, the developmental timing and chronicity of adversities could be examined as potential moderators of both the direct and indirect effects of ACEs on psychopathology (Gee, 2021).

## Conclusion

This study contributes to a deeper understanding of how different domains of childhood adversity influence emerging adult mental health via distinct emotional and cognitive mechanisms. Family maltreatment and peer victimization operate through multiple overlapping pathways, i.e. perseverative thinking, threat interpretation bias and emotional reactivity, whereas sexual abuse may affect psychopathology through different, less well-understood routes. These findings underscore the importance of tailoring interventions based on the specific adversity domains experienced and the observed difficulties with emotional and social information processing. Targeting perseverative thinking and threat-related cognitive processes may be particularly effective in reducing the impact of ACEs on psychopathology in emerging adulthood.

## Declaration of generative AI and AI-assisted technologies in the writing process

During the writing process the first author used OpenAI/ChatGPT to improve the readability and language of the manuscript. After using this tool/service, the author reviewed and edited the content as needed and takes full responsibility for the content of the published article.

## Declaration of competing interest

All authors declare that they have no conflict of interest regarding this paper.

## Appendix Supplementary materials

Image, application 1
