# Supplementary material for: Perseverative thinking, threat interpretation bias, and emotional reactivity as mediators between adverse childhood experience domains and psychopathology: A longitudinal mediation study in a cohort of Swiss emerging adults
Source: Int J Clin Health Psychol. 2025 Dec 11;25(4):100654. doi: 10.1016/j.ijchp.2025.100654 (PMC12756547; doi:10.1016/j.ijchp.2025.100654)
Supplement: Supplementary file 1 [file mmc1.docx]

# Supplemental Material 1: Details Factor Analyses

**Methods**

**Factor Analysis of Adverse Childhood Experiences (ACEs)**

A factor analytic approach was employed to derive latent factors of ACEs. First, an *Exploratory Factor Analysis* (EFA) was conducted on a random split-half of the sample (Split 1, *n* = 1,000) using Geomin rotation and the robust Weighted Least Squares Mean and Variance adjusted estimator (WLSMV) appropriate for categorical data. The three-, four-, and five-factor models shoed acceptable to good goodness-of-fit indices (see Table S1). The three-factor EFA model resulted in a factor including all items of the CTQ and MACE, a second factor with the items for emotional neglect and sexual abuse and a third factor for peer victimization. The four- factor yielded well-defined, theoretically meaningful factors. The five-factor model did not contain well-defined factors, each with at least three indicators loading ≥ .50.

Based on the EFA findings, we conducted a *Confirmatory Factor Analysis (CFA)* for the four- factor model using the WLSMV estimator on the second split-half (Split 2, *n* = 934). The factor structure was consistent across both halves and the full sample. Therefore, CFA results are reported for the full sample (*N* = 1,934) to maximize power.

Model fit was assessed using the Comparative Fit Index (CFI), Tucker–Lewis Index (TLI), and Root Mean Square Error of Approximation (RMSEA). Although no single fit index should be interpreted in isolation, models demonstrating CFI and TLI values greater than .95 and RMSEA values close to .05 were considered to indicate good fit (Hu & Bentler, 1999).

**Results**

Table S1 describes the goodness of fit indices for the exploratory and the confirmatory factor analyses. Among several exploratory factor models, only the four-factor solution demonstrated acceptable model fit—CFI and TLI > .95, RMSEA < .07—and well-defined factors, each with at least three indicators loading ≥ .40 with no substantial cross-loadings on another factor (Brown, 2015).
**Table S1**

*Goodness of Fit Indices for the Exploratory and the Confirmatory Factor Analyses*

Chi Square df CFI TLI RMSEA RMSEA 90% CI

Exploratory factor analysis half 1 sample

| 3 factor model | 2758.61 | 525 | 0.94 | 0.93 | 0.065 | [0.06, 0.07] |
| --- | --- | --- | --- | --- | --- | --- |
| 4 factor model | 1856.55 | 492 | 0.96 | 0.95 | 0.053 | [0.05, 0.06] |
| 5 factor model | 1294.55 | 460 | 0.98 | 0.97 | 0.043 | [0.04, 0.05] |
| 6 factor model | 900.90 | 429 | 0.99 | 0.98 | 0.033 | [0.03, 0.04] |
| Confirmatory factor analysis half 2 sample | | | | | | |
| 4 factor model | 1975.16 | 588 | 0.96 | 0.96 | 0.049 | [0.47, 0.51] |
| Confirmatory factor analysis full sample | | | | | | |
| 4 factor model | 3516.02 | 588 | 0.96 | 0.95 | 0.051 | [0.05, 0.05] |
| Note: df degrees of freedom, CFI Comparative Fit Index; TLI Tucker–Lewis Index; RMSEA Root Mean Square Error of Approximation. | | | | | | |
|  |  |  |  |  |  |  |

The four-factor confirmatory factor analysis identified the following factors, with the items and item loadings described in Table S2:

The four-factor model of the CFA demonstrated good fit and identified the following factors, with the items and item loadings described in Table S2:

1) Family maltreatment, 18 items, including CTQ items for abuse and neglect as well as the MACE items for witnessing domestic violence (Cronbach’s α = .89, McDonald’s Ω = .96). As underlying source of covariance, the items on this factor share defining features such as a hostile or neglectful family environment that can include coercive or harsh parenting, family-level interpersonal violations, and/or deficit in care/concern by the parent(s).

2) Verbal and physical peer victimization, all respective 5 MACE items (Cronbach’s α =.86 McDonald’s Ω = .94). As underlying source of covariance, these items can share defining features of the social environments (e.g. classroom and school climates and policies) as well as peer groups processes that are characterized by dominance, power asymmetries, and peer aggression.

3) Sexual abuse, 6 items including all sexual abuse items of the respective CTQ scale and sexual abuse by peers (Cronbach’s α = .89, McDonald’s Ω = .97). As underlying source of covariance, the defining feature of this factor is the sexual nature of the experiences, regardless of perpetrator or context. Unlike the first two factors, these items cluster based on the specific type of violation rather than the family or peer context. Distinct phenomenology and relational/behavioural features of sexual abuse involve sexual boundary violations, coercion and often secrecy and betrayal dynamics and that are qualitatively different from physical abuse, emotional hostility, or neglect. Furthermore, child sexual abuse shows associations with specific outcomes such as sexual revictimization and sexual health consequences (Werner et al. (2016).

A fourth factor comprised all positively phrased and recoded items (i.e., all CTQ emotional neglect items and two CTQ physical neglect items: “Got taken care of” and “Got taken to the doctor when needed”). This indicates that the factor functioned as a method factor (Podsakoff et al., 2024). Because it was also strongly correlated with the family maltreatment factor (*r* = .80), it was excluded from the analyses, while still contributing to the estimation of the ACE factor model.

**Table S2**

*Standardised Factor Loadings of the Confirmatory Four-Factor Model*

| Item | CTQ/MACE subscale | Estimate | S.E. |
| --- | --- | --- | --- |
|  |  |  |  |
| *Factor 1: Family Maltreatment* |  |  |  |
| Called names by family | EA1 | 0.78 | 0.01 |
| Parents wished was never born | EA2 | 0.82 | 0.01 |
| Family said hurtful things | EA3 | 0.82 | 0.01 |
| Felt hated by family | EA4 | 0.85 | 0.01 |
| Was emotionally abused | EA5 | 0.88 | 0.01 |
| Hit hard enough by family member to leave bruises ^a^ | PA1 | 0.81 | 0.02 |
| Hit hard enough by family member to see doctor ^a^ | PA2 | 0.73 | 0.05 |
| Beaten with hard objects ^a^ | PA3 | 0.70 | 0.02 |
| Was physically abused ^a^ | PA4 | 0.83 | 0.02 |
| Hit badly enough to be noticed by teacher, neighbour or doctor ^a^ | PA5 | 0.85 | 0.03 |
| Not enough to eat ^a^ | PN1 | 0.59 | 0.04 |
| Parents were high or drunk ^a^ | PN3 | 0.61 | 0.04 |
| Wore dirty clothes ^a^ | PN4 | 0.54 | 0.04 |
| Parents arguing violently | WV1 | 0.66 | 0.02 |
| Witnessed physical violence between parents | WV2 | 0.81 | 0.01 |
| Witnessed parent got injured by other parent | WV3 | 0.91 | 0.02 |
| Witnessed physical violence towards sibling | WV4 | 0.64 | 0.02 |
| Witnessed sibling got injured | WV5 | 0.77 | 0.03 |
|  |  |  |  |
| F*actor 2: Peer Victimization* |  |  |  |
| Called names by peers | PR1 | 0.88 | 0.01 |
| Verbal abuse by peers | PR2 | 0.91 | 0.01 |
| Excluded by peers | PR3 | 0.86 | 0.01 |
| Hit by peers | PR4 | 0.81 | 0.01 |
| Injured by peers | PR5 | 0.93 | 0.02 |
|  |  |  |  |
| *Factor 3: Sexual Abuse* |  |  |  |
| Was touched sexually a | SA1 | 0.94 | 0.01 |
| Threatened if did not do something sexual ^a^ | SA2 | 0.95 | 0.03 |
| Made to do sexual things ^a^ | SA3 | 0.95 | 0.01 |
| Was sexually harassed ^a^ | SA4 | 0.94 | 0.01 |
| Was sexually abused ^a^ | SA5 | 0.96 | 0.01 |
| Peer(s) forced sexual activity against will ^a^ | PR6 | 0.81 | 0.04 |
| *Factor 4: Positively Worded Items /Neglect* |  |  |  |
| Made to feel important by family member (R) | EN1 | 0.83 | 0.01 |
| Felt loved (R) ^a^ | EN2 | 0.90 | 0.01 |
| Was looked out for by family (R) | EN3 | 0.92 | 0.01 |
| Family felt close (R) | EN4 | 0.89 | 0.01 |
| Family was source of strength (R) | EN5 | 0.91 | 0.01 |
| Got taken care of by family (R) | PN2 | 0.83 | 0.01 |
| Got taken to doctor (R) ^a^ | PN5 | 0.57 | 0.03 |

Note: All p-values < .001; CTQ: Childhood Trauma Questionnaire; MACE: Maltreatment and Abuse Chronology of Exposure Scale; EN: Emotional neglect; EA: Emotional abuse; PN: Physical neglect; PA: Physical abuse; SA: Sexual abuse; WV: Witnessing domestic violence; PV: Peer victimization. (R) positively phrased and recoded item.
^a^ Responses in the highest categories were collapsed so that each category had at least 20 participants. For details about answer frequencies, see Marmet et al. (2024).

As factor 1 comprised all positively phrased and recoded items (i.e., all emotional neglect items and one physical neglect item: “being taken to the doctor when needed”) and was strongly correlated with the family maltreatment factor (*r* = .80), suggesting it functioned as a method factor. As such, while this factor informed the estimation of the ACE factor model, it was excluded from further mediation analyses.

**References**

Brown, T. A. (2015). *Confirmatory Factor Analysis for Applied Research, Second Edition*. Guilford Publications.

Hu, L., & Bentler, P. M. (1999). Cutoff criteria for fit indexes in covariance structure analysis: Conventional criteria versus new alternatives. *Structural Equation Modeling: A Multidisciplinary Journal*, *6*(1), 1–55. <https://doi.org/10.1080/10705519909540118>

Werner, K. B., McCutcheon, V. V., Challa, M., Agrawal, A., Lynskey, M. T., Conroy, E., ... & Nelson, E. C. (2016). The association between childhood maltreatment, psychopathology, and adult sexual victimization in men and women: results from three independent samples. *Psychological medicine*, *46*(3), 563-573).

**Supplemental Material 2: Details Mediation Models**

**Proximal, Sustained, and Distal Indirect Effects of ACEs on Psychopathology at W2 and W3**

*Single mediation models*

Associations between family maltreatment and peer victimization and subsequent psychopathology at W2 and W3 were mediated by emotional reactivity, perseverative thinking, and threat interpretation bias, as each exhibited small but statistically significant indirect effects. For sexual abuse, only threat interpretation bias functioned as a significant mediator, also with a small effect size. Rejection sensitivity was not confirmed as a mediator. This supplement details the proximal, sustained, and distal indirect effects exhibited by these mediators. Table 4 in the main text provides the details for the direct and indirect paths for psychopathology at W3 described below. Table S3 in this supplement presents the direct and indirect effects for psychopathology at W2.

Perseverative thinking demonstrated significant proximal (X → M1 → Y2 → Y3) and sustained (X → M1 → M2 → Y3) indirect effects for family maltreatment and peer victimization, explaining subsequent psychopathology. Furthermore, the only distal (X → M2 → Y3) indirect effect in all mediation models was found for perseverative thinking linking peer victimization to subsequent psychopathology.

Threat interpretation bias exhibited proximal and marginal sustained indirect effects for the family maltreatment and peer victimization factors. Moreover, threat interpretation bias was the only mediator that linked sexual abuse to psychopathology with a proximal indirect effect only.

Emotional reactivity showed only a proximal effect via the X → M1 → Y2 → Y3 path for family maltreatment and peer victimization, indicating that emotional reactivity has only a short-term effect.

*Multiple Mediation Model*

Indirect effects were only observed for family maltreatment and peer victimization, not for sexual abuse.

Perseverative thinking demonstrated a significant sustained indirect effect on psychopathology at W3 (X → M1 → M2 → Y3) for family maltreatment and peer victimization. Additionally, results confirmed the distal indirect effect via the X → M2 → Y3 path for peer victimization in the single mediator model, but not the proximal effects in the single mediator models.

Threat interpretation bias exhibited significant proximal indirect effects on psychopathology at W2 for both family maltreatment and peer victimization via the X → M1 → Y2 path. These proximal indirect effects were confirmed for psychopathology at W3 via the X → M1 → Y2 → Y3 path. However, the sustained indirect effects in the single mediator model were not confirmed.

Emotional reactivity showed a significant proximal indirect effect for peer victimization on heightened psychopathology via the path X → M1 → Y2. The proximal indirect effect on psychopathology at W2 for family maltreatment in the single mediator model was not confirmed. Conversely, emotional reactivity showed a significant negative sustained indirect effect for both family maltreatment and peer victimization, predicting lower psychopathology at W3 via the X → M1 → M2 → Y3 path.

**Table S3**

*Longitudinal Mediation Models: Direct and Indirect Effects of Single Mediators on Psychopathology at Wave 2*

| Paths and effects for W2 | *Family maltreatment* | | | | | | *Peer victimization* | | | | | | | | *Sexual abuse* | | | | | | | |  |  |  |
| --- | --- | --- | --- | --- | --- | --- | --- | --- | --- | --- | --- | --- | --- | --- | --- | --- | --- | --- | --- | --- | --- | --- | --- | --- | --- |
|  | *est.* | *95% CI* | | *p* | | *std.* | *est.* | | *95% CI* | | | *p* | *std.* | | *est.* | | *95% CI* | | | *p* | | *std.* |  |  |  |
| *Perseverative thinking* |  |  | |  | |  |  | |  | | |  |  | |  | |  | | |  | |  |  |  |  |
| Overall indirect effect | **0.01** | **[0.00, 0.02]** | | **.019** | | **n/a** | **0.01** | | **[0.00, 0.01]** | | | **.025** | **n/a** | | 0.00 | | [0.00, 0.01] | | | .269 | | n/a |  |  |  |
| x->M1 ->Y2 | **0.01** | **[0.00, 0.02]** | | **.019** | | **0.02** | **0.01** | | **[0.00, 0.01]** | | | **.025** | **0.01** | | 0.00 | | [0.00, 0.01] | | | .269 | | 0.00 |  |  |  |
| Overall direct effect | **0.14** | **[0.08, 0.19]** | | **<.001** | | **n/a** | **0.12** | | **[0.08, 0.16]** | | | **<.001** | **n/a** | | 0.06 | | [-0.01, 0.13] | | | .090 | | n/a |  |  |  |
| x->Y1 -> Y2 | **0.08** | **[0.06, 0.11]** | | **<.001** | | **0.12** | **0.06** | | **[0.04, 0.08]** | | | **<.001** | **0.09** | | **0.04** | | **[0.02, 0.07]** | | | **.001** | | **0.05** |  |  |  |
| x-> Y2 | **0.05** | **[0.01, 0.10]** | | **.033** | | **0.08** | **0.06** | | **[0.02, 0.10]** | | | **.002** | **0.09** | | 0.01 | | [-0.05, 0.07] | | | .654 | | 0.02 |  |  |  |
| Total effect | **0.15** | **[0.10, 0.20]** | | **<.001** | | **0.21** | **0.13** | | **[0.09, 0.17]** | | | **<.001** | **0.20** | | 0.06 | | [-0.01, 0.13] | | | .079 | | 0.07 |  |  |  |
| % total by indirect | **7.35** |  | |  | |  | **5.47** | |  | | |  |  | | 4.92 | |  | | |  | |  |  |  |  |
| *Emotional reactivity* |  |  | |  | |  |  | |  | | |  |  | |  | |  | | |  | |  |  |  |  |
| Overall indirect effect | **0.01** | **[0.00, 0.02]** | | **.003** | | **n/a** | **0.02** | | **[0.01, 0.03]** | | | **<.001** | **n/a** | | 0.00 | | [0.00,0.01] | | | .571 | | n/a |  |  |  |
| x->M1 ->Y2 | **0.01** | **[0.00, 0.02]** | | **.003** | | **0.02** | **0.02** | | **[0.01, 0.03]** | | | **<.001** | **0.03** | | 0.00 | | [0.00, 0.01] | | | .571 | | 0.00 |  |  |  |
| Overall direct effect | **0.14** | **[0.09, 0.19]** | | **<.001** | | **n/a** | **0.11** | | **[0.07, 0.15]** | | | **<.001** | **n/a** | | 0.06 | | [-0.01, 0.12] | | | .100 | | n/a |  |  |  |
| x->Y1 -> Y2 | **0.09** | **[0.06, 0.11]** | | **<.001** | |  | **0.07** | | **[0.05, 0.09]** | | | **<.001** | **0.10** | | **0.05** | | **[0.02, 0.07]** | | | **.001** | | **0.05** |  |  |  |
| x-> Y2 | **0.05** | **[0.00, 0.10]** | | **.037** | | **0.07** | **0.05** | | **[0.01, 0.08]** | | | **.010** | **0.07** | | 0.01 | | [-0.05, 0.07] | | | .756 | | 0.01 |  |  |  |
| Total effect | **0.15** | **[0.10, 0.20]** | | **<.001** | | **0.21** | **0.13** | | **[0.09, 0.17]** | | | **<.001** | **0.20** | | 0.06 | | [-0.01, 0.13] | | | .096 | | 0.07 |  |  |  |
| % total by indirect | **6.76** |  | |  | |  | **13.18** | |  | | |  |  | | 3.51 | |  | | |  | |  |  |  |  |
| *Threat interpretation bias* |  |  | |  | |  |  | |  | | |  |  | |  | |  | | |  | |  |  |  |  |
| Overall indirect effect | **0.02** | **[0.01, 0.03]** | | **<.001** | | **n/a** | **0.02** | | **[0.01, 0.03]** | | | **<.001** | **n/a** | | **0.01** | | **[0.00, 0.02]** | | | **.03** | | **n/a** |  |  |  |
| x->M1 ->Y2 | **0.02** | **[0.01, 0.03]** | | **<.001** | | **0.03** | **0.02** | | **[0.01, 0.03]** | | | **<.001** | **0.03** | | **0.01** | | **[0.00, 0.02]** | | | **.03** | | **0.01** |  |  |  |
| Overall direct effect | **0.13** | **[0.09, 0.18]** | | **<.001** | | **n/a** | **0.11** | | **[0.08, 0.15]** | | | **<.001** | **n/a** | | 0.05 | | [-0.01, 0.11] | | | .19 | | n/a |  |  |  |
| x->Y1 -> Y2 | **0.08** | **[0.06, 0.10]** | | **<.001** | | **0.12** | **0.06** | | **[0.04, 0.08]** | | | **<.001** | **0.09** | | **0.04** | | **[0.02, 0.07]** | | | **.001** | | **0.05** |  |  |  |
| x-> Y2 | **0.05** | **[0.00, 0.10]** | | **.040** | | **0.07** | **0.05** | | **[0.01, 0.09]** | | | **.006** | **0.08** | | 0.00 | | [-0.06, 0.06] | | | .938 | | 0.00 |  |  |  |
| Total effect | **0.15** | **[0.11, 0.20]** | | **<.001** | | **0.22** | **0.13** | | **[0.10, 0.17]** | | | **<.001** |  | | 0.05 | | [0.00, 0.12] | | | .121 | | 0.06 |  |  |  |
| % total by indirect | **12.75** |  |  | |  | | **13.85** | | | |  | | | **16.67** | | | | |  | |  | | |  |  |
|  |  |  |  | |  | |  |  | |  |  | | |  | |  | |  |  | |  | | |  |  |
| *Rejection sensitivity* |  |  |  | |  | |  |  | |  |  | | |  | |  | |  |  | |  | | |  |  |
| Overall indirect effect | 0.01 | [0.00, 0.01] | | .162 | | n/a | 0.00 | | [0.00, 0.01] | | | .156 | n/a | | 0.00 | | [-0.01, 0.00] | | | .604 | | n/a |  |  |  |
| x->M1 ->Y2 | 0.01 | [0.00, 0.01] | | .162 | | 0.01 | 0.00 | | [0.00, 0.01] | | | .156 | 0.01 | | 0.00 | | [-0.01, 0.00] | | | .604 | | 0.00 |  |  |  |
| Overall direct effect | **0.14** | **[0.09, 0.20]** | | **<.001** | | **n/a** | **0.13** | | **[0.08, 0.17]** | | | **<.001** | **n/a** | | 0.06 | | [-0.01, 0.13] | | | .093 | | n/a |  |  |  |
| x->Y1 -> Y2 | **0.09** | **[0.07, 0.11]** | | **<.001** | | **0.13** | **0.07** | | **[0.05, 0.09]** | | | **<.001** | **0.10** | | **0.05** | | **[0.02, 0.08]** | | | **.001** | | **0.06** |  |  |  |
| x-> Y2 | **0.05** | **[0.01, 0.10]** | | **.033** | | **0.08** | **0.06** | | **[0.02, 0.10]** | | | **.002** | **0.09** | | 0.01 | | [-0.05, 0.07] | | | .737 | | 0.01 |  |  |  |
| Total effect | **0.15** | **[0.10, 0.20]** | | **<.001** | | **0.21** | **0.13** | | **[0.09, 0.17]** | | | **<.001** | **0.20** | | 0.06 | | [-0.01, 0.13] | | | .099 | | 0.07 |  |  |  |
| % total by indirect | 3.40 |  |  | |  | |  | 3.08 | |  |  | | | 1.75 | | | | |  | |  | | |  |  |

Note: Significant results are shown in bold. Results are controlled for confounders. X: Adversity domain; M1: Mediator at Wave 1;

M2: Mediator at Wave 2; Y1: Psychopathology (BSI-18) at Wave 1; Y2: Psychopathology (BSI-18) at Wave 2; Y3: Psychopathology (BSI-18) at Wave 3;

est.: unstandardised estimate; std.: standardised estimate.

**Table S4**

*Multiple Longitudinal Mediation Model: Direct Paths on Psychopathology*

| *Direct effects* | *Psychopathology (BSI-18) Wave 1* | | | | Psychopathology (BSI-18) Wave 2 | | | | Psychopathology (BSI-18) Wave 3 | | | |
| --- | --- | --- | --- | --- | --- | --- | --- | --- | --- | --- | --- | --- |
|  | *est.* | *95% CI* | *p* | std. | *est.* | *95% CI* | *p* | std. | *est.* | *95% CI* | *p* | std. |
| Family maltreatment | **0.19** | **[0.14, 0.23]** | **<.001** | **0.26** | **0.06** | **[0.01, 0.11]** | **.027** | **0.08** | 0.01 | [-0.04, 0.06] | .776 | 0.01 |
| Peer victimization | **0.14** | **[0.10, 0.18]** | **<.001** | **0.20** | **0.06** | **[0.02, 0.09]** | **.004** | **0.09** | 0.04 | [-0.01, 0.08] | .081 | 0.06 |
| Sexual abuse | **0.10** | **[0.04, 0.16]** | **.001** | **0.11** | 0.01 | [-0.05, 0.07] | .757 | 0.01 | **0.09** | **[0.03, 0.15]** | **.005** | **0.11** |
| Perseverative thinking W1/W2^a^ | -- | -- | -- | -- | 0.02 | [-0.02, 0.06] | .385 | 0.03 | **0.07** | **[0.03, 0.12]** | **.001** | **0.11** |
| Threat interpretation bias W1/W2 ^a^ | -- | -- | -- | -- | **0.04** | **[0.00, 0.07]** | **.028** | **0.07** | 0.02 | [-0.01, 0.06] | .232 | 0.05 |
| Emotional reactivity W1/W2 ^a^ | -- | -- | -- | -- | 0.02 | [-0.02, 0.06] | .262 | 0.03 | **-0.05** | **[-0.10, -0.01]** | **.020** | **-0.07** |

Note: Significant results are shown in bold. Results are controlled for confounders.

^a^ Lagged paths from mediator at Wave 1 on psychopathology at Wave 2 and mediator Wave 2 on psychopathology at Wave 3; est.: unstandardised estimate; std.: standardised estimate.
